# Phylogenetic and recombination analysis of the herpesvirus genus varicellovirus

**DOI:** 10.1186/s12864-017-4283-4

**Published:** 2017-11-21

**Authors:** Aaron W. Kolb, Andrew C. Lewin, Ralph Moeller Trane, Gillian J. McLellan, Curtis R. Brandt

**Affiliations:** 1 Department of Ophthalmology and Visual Sciences, School of Medicine and Public Health, University of Wisconsin-Madison, 550 Bardeen Laboratories, 1300 University Ave., Madison, WI 53706 USA; 2 Department of Surgical Sciences, School of Veterinary Medicine, University of Wisconsin-Madison, Madison, WI USA; 3 McPherson Eye Research Institute, University of Wisconsin-Madison, Madison, WI USA; 4 Medical Microbiology and Immunology, School of Medicine and Public Health, University of Wisconsin-Madison, Madison, WI USA

**Keywords:** Varicellovirus, Genome, Herpes, Veterinary, Phylogeny, Virus, Recombination

## Abstract

**Background:**

The varicelloviruses comprise a genus within the alphaherpesvirus subfamily, and infect both humans and other mammals. Recently, next-generation sequencing has been used to generate genomic sequences of several members of the *Varicellovirus* genus. Here, currently available varicellovirus genomic sequences were used for phylogenetic, recombination, and genetic distance analysis.

**Results:**

A phylogenetic network including genomic sequences of individual species, was generated and suggested a potential restriction between the ungulate and non-ungulate viruses. Intraspecies genetic distances were higher in the ungulate viruses (pseudorabies virus (SuHV-1) 1.65%, *bovine herpes virus type 1* (BHV-1) 0.81%, *equine herpes virus type 1* (EHV-1) 0.79%, *equine herpes virus type 4* (EHV-4) 0.16%) than non-ungulate viruses (*feline herpes virus type 1* (FHV-1) 0.0089%, *canine herpes virus type 1* (CHV-1) 0.005%, varicella-zoster virus (VZV) 0.136%). The G + C content of the ungulate viruses was also higher (SuHV-1 73.6%, BHV-1 72.6%, EHV-1 56.6%, EHV-4 50.5%) compared to the non-ungulate viruses (FHV-1 45.8%, CHV-1 31.6%, VZV 45.8%), which suggests a possible link between G + C content and intraspecies genetic diversity. Varicellovirus clade nomenclature is variable across different species, and we propose a standardization based on genomic genetic distance. A recent study reported no recombination between sequenced FHV-1 strains, however in the present study, both splitstree, bootscan, and PHI analysis indicated recombination. We also found that the recently sequenced Brazilian CHV-1 strain BTU-1 may contain a genetic signal in the UL50 gene from an unknown varicellovirus.

**Conclusion:**

Together, the data contribute to a greater understanding of varicellovirus genomics, and we also suggest a new clade nomenclature scheme based on genetic distances.

**Electronic supplementary material:**

The online version of this article (10.1186/s12864-017-4283-4) contains supplementary material, which is available to authorized users.

## Background

The *Varicellovirus* genus is part of the larger alphaherpesvirus subfamily which includes herpes simplex viruses 1 and 2, as well as Marek’s disease virus. Like other alphaherpesviruses, varicelloviruses typically infect epithelial surfaces, and most appear to be neurotropic, establishing latency in neurons [1–9]. The first varicellovirus to be clinically described as a unique disease was varicella zoster virus (VZV), the causative agent of chickenpox and shingles in 1767 [10]. Numerous other varicelloviruses have been identified, including pseudorabies virus/SuHV-1 (Aujeszky’s disease) in pigs, BHV-1 (*bovine herpes virus type 1*; infectious bovine rhinotracheitis; IBR), EHV-1 (*equine herpes virus type 1*; epidemic abortion and myeloencephalopathy in horses), EHV-4 (*equine herpes virus type 4*; equine rhinopneumonitis), FHV-1 (*feline herpes virus type 1*; feline rhinotracheitis), and CHV-1 (*canine herpes virus type 1*; fading puppy syndrome). These viruses have significant impact on livestock and companion animals. Due to high transmissibility and virulence, pseudorabies virus and EHV-1 are both classified by the United States Department of Agriculture (USDA) as reportable diseases [11]. Vaccines have been developed against several varicelloviruses, including VZV, pseudorabies virus, BHV-1, EHV-1, EHV-4, FHV-1, CHV-1, which have been effective at reducing morbidity and mortality [12–22]. Pseudorabies vaccination and eradication efforts in the United States have been effective, with the country declared disease free in 2004. The USA and Canada have also enacted BHV-1 control programs, and several European countries have successfully eradicated the disease [23]. Despite vaccination and control efforts, many of these diseases continue to negatively affect humans and animals worldwide.

The first complete sequence of a varicellovirus, (VZV) was reported in 1986 [24], followed by several others [25–27] . The advent of next-generation sequencing (NGS) has revolutionized genomics, and has allowed additional varicellovirus species and sub-strains to be sequenced. The first comprehensive computational multigene phylogenetic analysis of the three main herpes virus subfamilies was a major step forward in cementing the basic structure for *Alphherpesvirinae*, including varicelloviruses [28]. As increasing numbers of viral strains have been sequenced, full genome phylogenetic and recombination analysis of VZV, SuHV-1, EHV-1, and EHV-4 have been performed [29–32].

The genetic code is degenerate, resulting in most amino acids being encoded by multiple codons. The usage of some codons and not others for an amino acid is often not random, and is called codon usage bias [33]. Codon usage and mutational bias analysis has been examined in several viruses, including phages, canine parvovirus, Japanese encephalitis virus, rabies, Zika virus, herpesviruses, and other vertebrate DNA viruses [34–40]. Shackelton et al. [39], showed that codon usage bias is strongly linked with genomic G + C content. The previous analysis of codon usage in herpesviruses [38] found strong codon bias in the SuHV-1 and BHV-5 viruses, both high G + C viruses. While the earlier herpesvirus study [38] included several varicelloviruses, additional viruses have now been sequenced, and a more inclusive analysis is now possible.

The goal of the current study was to perform a genome based comprehensive phylogenetic, genetic distance, and recombination analysis of the varicellovirus subfamily. Unique findings reported here are a phylogenetic stricture between ungulate herpesviruses and the remaining species, a possible link between genomic G + C content and intraspecies distance, the identification of recombination amongst FHV-1 strains, and results suggesting that the Brazilian CHV-1 strain BTU-1 may be a recombinant between CHV-1 and an unknown varicellovirus. We also propose a *Varicellovirus* genus clade nomenclature standardization based on genetic distance.

## Methods

### Genomic multiple sequence alignments

For phylogenetic and distance analysis, currently available *Varicellovirus* genus genomic sequences were downloaded from NCBI, and are cataloged in Additional file 1: Table S1. The first generated alignment was of the *Varicellovirus* genus as a whole, using one strain from each of the viral species, as well as an outgroup, *anatid herpes virus type 1* (AnHV-1). AnHV-1 was chosen as an outgroup because the AnHV-1 is an alphaherpesvirus, and the genome is annotated in a similar fashion to the varicelloviruses. The varicelloviruses and the AnHV-1 outgroup virus have similar genome annotation and gene synteny, with the exception of pseudorabies virus. In pseudorabies virus, the UL27 to UL44 genes are inverted. Prior to genome alignment, the UL27-UL44 genome segment of pseudorabies virus was inverted by reverse complemention in order to generate a gene order similar to the other varicellovirueses. MAFFT v2.66 [41, 42] was utilized to generate the alignment using the FFT-NS-1 method. The subsequent genomic multiple sequence alignment was manually inspected for quality. Areas of the alignment that appeared to be of poor quality were realigned in Mega 6 [43] using ClustalW.

Additional intraspecies genomic alignments of BHV-1, CHV-1, EHV-1, EHV-4, FHV-1, SuHV-1, and VZV, were generated with and without outgroups using MAFFT v2.66. The outgroups for the intraspecies alignments were chosen based on low genetic distance, in other words, the closest known relative. Thus, for EHV-1 analysis, EHV-8 was chosen as the outgroup, with the remaining analyzed species/outgroup combinations being; BHV-1/outgroup BHV-5, EHV-4/outgroup EHV-1, SuHV-1/outgroup BHV-1, FHV-1/outgroup CHV-1, CHV-1/outgroup FHV-1, and VZV/outgroup CeHV-9. All of the genomic alignments generated in thus study are available for download at http://sites.ophth.wisc.edu/brandt/.

### Genetic distance and genomic G + C content calculations

The mean maximum likelihood (ML) distances for each alignment were calculated using the Mega 6 package. For the genetic distance analysis, pairwise gap deletion rather than complete deletion of gaps was used, as complete deletion of alignment gaps may exclude valuable phylogenetic data, and could result in an underestimation of distance. To calculate overall genome G + C content, an online calculator found at http://www.endmemo.com/bio/gc.php was used.

### Phylogenetic and recombination analysis

For phylogenetic maximum likelihood and network analysis, intraspecies genomic alignments of BHV-1, CHV-1, EHV-1, EHV-4, FHV-1, SuHV-1, VZV, and total sequenced varicelloviruses were generated using duck enteritis virus (AnHV-1) as the outgroup as described above. Maximum likelihood phylogenetic analysis was performed on the genomic alignments containing an outgroup using the RAxMLGUI package [44] with the GTRCAT + I model and 500 bootstrap replicates.

Phylogenetic networks for BHV-1, CHV-1, EHV-1, EHV-4, FHV-1, SuHV-1, VZV, and total sequenced varicelloviruses were generated with Splitstree 4 [45] using multiple sequence alignments and AnHV-1 as the outgroup. Splitstree was also used to calculate the pairwise homoplasy index (PHI) statistical test [46] for recombination. Jmodeltest2 [47] was used to identify the optimal substitution model settings for each individual phylogenetic network. RDP4 [48] recombination analysis performed genomic multiple sequence alignments without outgroups using the Jin and Nei substitution model with the following parameters: 1500 bp window, 750 bp step size, and 200 bootstrap replicates.

### Determining shared clade cut-off values between BHV-1, EHV-1, and SuHV-1

Shared clade Cut-off values between BHV-1, EHV-1, and SuHV-1 were determined by first generating a histogram of pairwise *p*-distances and corresponding frequencies for each virus species, similar to the study performed by Grau-Roma et el with *porcine circovirus type 2* (PCV2) [49].The pairwise *p*-distances for each species were calculated using Mega 6, and multiple sequence alignments without outgroups. Histograms of frequency vs *p*-distance for BHV-1, EHV-1, and SuHV-1, and subsequent data were generated using R (version 3.4.2 using the ggplot2 package). An initial shared cut-off of 0.01 was established by examining the upper and lower bounds of the two main groups in the three histograms. This intial cut-off was further evaluated, using a variance analysis framework, where variance between and within groups was examined. For each potential cut-off value, we calculated the following quantities for the *p*-distances for each virus:

For each potential cut-off value, we calculate the following quantities:

*SS*_between_ = ∑*f*_*ij*_·(group mean *j* − overall mean),^2^

*SS*_within_ = ∑*f*_*ij*_·(p-distance *i* − overall),^2^

where.

group mean_*j*_ = mean *p*-distance in group_j_, *j* = 1, 2,

overall mean = overall mean *p*-distance,

*p*-distance *i* = the *i* ^*th*^ *p*-distance,

*f*_*ij*_ = the frequency of the *i* ^*th*^ *p*-distance in the *j* ^*th*^ group.

Finally, we calculate ratio

$$ \mathit{\mathsf{F}}=\frac{{\mathit{\mathsf{SS}}}_{\mathit{\mathsf{between}}}}{{\mathit{\mathsf{SS}}}_{\mathit{\mathsf{within}}}/\left({\mathit{\mathsf{N}}}_{\mathit{\mathsf{j}}}-\mathsf{2}\right)} $$

where *N*_*j*_ is the total number of observations in group *j*, or in terms of frequencies, *N*_*j*_ = ∑_*i*_ *f*_*ij*_. We next wanted to determine the cut-off which maximized the quantity across the groups, by first plotting the *F* values for each of the three graphs (Figure S1). We next restricted the cut-off values where the *p*-values for the different viruses was divided into two groups. For example, this means that values larger than 0.012 were discarded as no such distances were found in the BHV-1 virus group. The values within each graph were rescaled 0 to 1 in order to make each virus of equal value, and the sum of the curves maximized. To examine how the *F* measure corresponded to the frequency distributions, the value of rescaled *F* was overlayed. The point at which the sum of the rescaled *F* values attains it’s maximum, was chosen as the cut-off value.

### Codon usage analysis

To investigate codon usage in the *Varicellovirus* genus as a whole, the effective number of codons (ENC) was calculated for the US1 (ICP22), UL30 DNA polymerase, and glycoprotein H genes from each varicellovirus species. These genes represent one member of the α (immediate-early), β (early), and γ (late) gene classes. The ENC value is a measure of how much the codon usage of a gene deviates from the equal usage of synonymous codons [50]. ENC values range from 20 to 61, with 20 indicating maximum bias, with one codon used from each synonymous codon group, to 61 indicating no codon usage bias. The ENC values for the varicellovirus US1, UL30, and gH genes were calculated using DnaSP (v5) [51]. In addition to the ENC values, GC3s values were calculated using DnaSP, while the GC1 and GC2 values were obtained using in the online calculator http://genomes.urv.es/CAIcal/. The GC12 values were calculated using Microsoft Excel. ENC-GC3s plots were generated using SigmaPlot v.11 . In the ENC-GC3s plots, if the plotted values are located on or near the standard curve, then codon usage is constrained only by G + C mutation bias. However, the greater the plotted values deviate from the standard curve, the more additional factors such as natural selection may influence the bias.

Neutral evolution plots (GC12s vs GC3s) were generated to examine the contribution of mutational pressure and natural selection. Sigmaplot v.11 was used to generate the plots, as well as for the linear regression statistical analysis.

## Results and discussion

### Nomenclature standardization

The nomenclature designation for varicellovirus species strains and intraspecies clades is somewhat variable, with some, such as bovine herpesvirus 1 strains given a BHV-1.1 or 1.2 designation [52], while VZV clades are given a simple numeral designations (1–6) [29]. As such, a clade nomenclature standardization across varicelloviruses may be useful, and we are proposing a common nomenclature system based on genetic distances. The International Committee on Taxonomy of Viruses (ICTV) does not provide guidelines in defining taxonomic clades below species level [53], and it is within the purview of interested groups to do so. Genetic distances have been previously used as a basis for nomenclature systems in H5N1 avian influenza [54] and porcine circovirus type 2 [55, 56]. The genetic distance based nomenclature system we are proposing would preserve classic BHV-1 1.1 and 1.2 clade designations, as well the varicella zoster virus (VZV) numerical designations. Within each species, clade/group distances greater than 1% would be designated by a 1.1, 1.2, ... numbering as seen in BHV-1 [52]. Between clade/group distances of less than 1% would result in a numerical format (i.e. 1, 2, 3…) as has been consistently used with VZV [29]. Under this system, it would be possible to have two distant clades given a 1.x designation, with less distant subclades designated numerically.

**Fig. 1:**
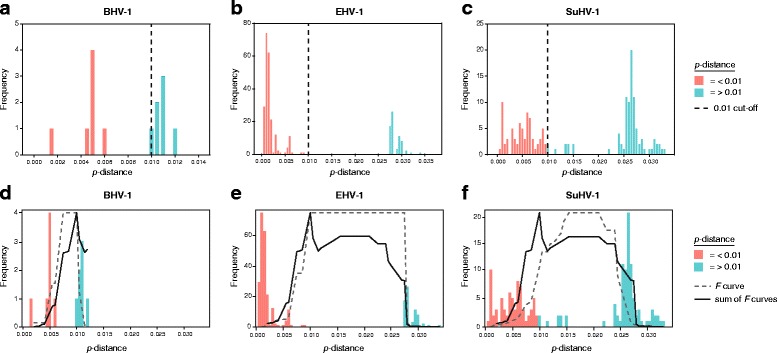
Establishing a shared phylogenetic clade cut-off value for BHV-1, EHV-1, and SuHV-1. To establish and initial cut-off value, pairwise *p*-distance values were calculated for BHV-1, EHV-1, and SuHV-1 using multiple sequence alignments. Frequency vs. p-distance histograms were generated for each of the three viruses (**a, b** and **c**). An initial cut-off of 0.01 was chosen, based on a shared *p*-distance value that divides the observed *p*-distances into two groups simultaneously for all three viruses (vertical dotted line; panels **a, b**, and **c**). Groups with *p*-distances <0.01 are colored salmon, while groups with *p*-distances >0.01 are colored teal. To evaluate the validity of the initial cut-off, variance analysis was performed, where variation between groups and within groups was examined. Panels **e, f**, and **g** show the individual rescaled *F* curves (gray dotted line) for each of the three viruses, as well as the sum of the curves in black. It is important to note the individual rescaled *F* and *F* sum curves cannot be directly compared as they are scaled differently, and do not correspond to the y-axis values. The *F* values were rescaled so as not to weight one virus more than the rest. The sum of the rescaled *F* curves shows a peak at 0.01, which validates the initial cut-off. The values of the unscaled, and rescaled *F* values are located in Additional file 1: Tables S2, and S3 respectively

The 1% cut-off was determined by calculating a shared value for the BHV-1, EHV-1, and SuHV-1 viruses. BHV-1, EHV-1, and SuHV-1 were chosen, as these three viruses have the highest levels of intraspecies genetic distance of the varicelloviruses (detailed in the sections below). An initial cut-off of 0.01 was chosen, based on a shared *p*-distance value that divides the observed *p*-distances into two groups simultaneously for all three viruses (Fig. 1a, b, and c). Additional evaluation of the initial cut-off was performed using a variance analysis framework, where variation between groups and within groups was examined. Figure 1e, f, and g show the individual rescaled *F* curves (gray dotted line) for each of the three viruses, as well as the sum of the curves in black. The *F* values were rescaled so as not to weight one virus more than the rest so the curves do not directly overlap. The sum of the rescaled *F* curves shows a peak at 0.01, which validates the initial cut-off. The values of the unscaled, and rescaled *F* values are shown in Additional file 1: Tables S2, and S3 respectively.

### Phylogenetic network analysis of varicelloviruses

**Fig. 2:**
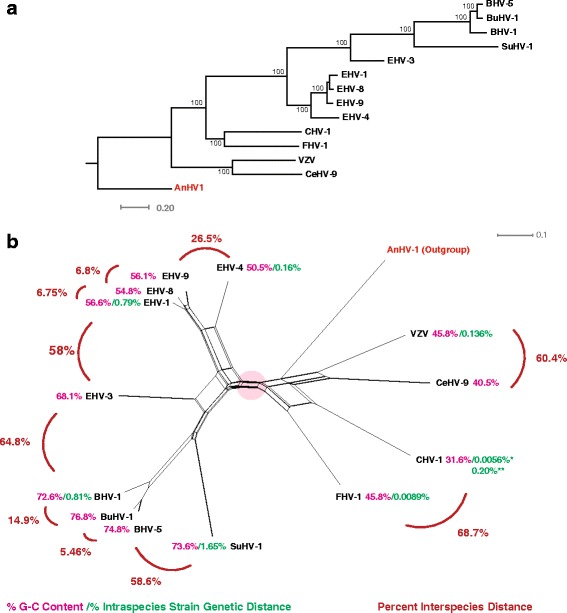
Phylogenetic, G + C content, intraspecies genetic distance analysis of the genome sequenced varicelloviruses. A maximum likelihood tree (**a**) was generated from a multiple sequence alignment (MSA) of the genome sequenced varicelloviruses using RAxML, and AnHV-1 as an outgroup. Bootstrap values over 65% are shown. **b** Splitstree was used to generate a phylogenetic network based on the varicellovirus (+ outgroup) MSA, and used settings obtained from jmodeltest2 (kimura 2-parameter, gamma = 2.3210, and *p*-inverse = 0.0890). The genome G + C composition percentages (pink) and intraspecies genetic distances (green; maximum composite likelihood, pairwise deletion) in panel **b** were calculated using http://www.endmemo.com/bio/gc.php and Mega 6, respectively. * denotes CHV-1 genetic distance based on three UK derived strains, while ** overall CHV-1 distance including stain BTU-1. The pink circle in the middle of the figure highlights a possible restriction between the two halves of the network

To investigate phylogenetic relationships between the sequenced varicelloviruses, the genomes of each species, along with the AnHV-1 outgroup genome were aligned. Both a maximum likelihood (ML) based tree (Fig. 2a) and phylogenetic network (Fig. 2b) were constructed. The ML whole genome based tree showed that CeHV-9 and VZV occupy a basal position in the genus, and the ungulate viruses share a node with bootstrap support of 100% (Fig. 2a). This result is fairly unremarkable and is similar to analysis performed using smaller sets of genes [57]. To assess the phylogenetic dissonance in the dataset, a phylogenetic network was also generated, and shows a similar basic topology with the ML tree. The network however suggests a stricture between the ungulates and non-ungulates, and is denoted by a pink circle (Fig. 2b). The stricture could be the result of low amounts of recombination between the two sides of the network, however, it may represent a bottleneck, or may be simply due to divergent phylogenetic signals. To determine if there was recombination within the network, the PHI statistical test for recombination was performed. The PHI test indicated statistically significant signals amongst the ungulate virus portion of the network, as well the non-ungulate portion, however, analysis of the network as a whole (minus outgroup) was not significant (Table 1). This lack of a significant result is likely due to the high amount of genetic distance within the dataset. The genomic distances between virus species are also shown in Fig. 2b to aid in data interpretation.

**Table 1:** Pairwise homoplasty index (PHI) statistic test *p*-values for recombination in the varicelloviruses

Virus	PHI *p*-value
VZV	< 0.001
CHV-1	0.3082
FHV-1	< 0.001
EHV-4	< 0.001
EHV-1	< 0.001
SuHV-1	< 0.001
BHV-1	< 0.001
*Varicellovirus* Genus	1.00
Ungulate Viruses	< 0.001
Primate and Carnivore Viruses	< 0.001

### Varicellovirus G + C content and interstrain genetic distance

The G + C content of each of the varicellovirus species was analyzed, with results shown in both Fig. 2b, and Table 2. All of the ungulate viruses had a G + C content above 50%, ranging from 50.5% in EHV-4 to 74.8% in BHV-5. The primate and carnivore viruses had G+C contents under 50%, and ranged from 31.6% in CHV-1 to 45.8% in both VZV and FHV-1. For each varicellovirus, where multiple strains have been sequenced, the overall intraspecies genetic distance for each species was calculated (Fig. 1b and Table 1). The results suggest a possible link between G + C content and intraspecies genetic distance with higher G + C content and genetic distance in the ungulate viruses (SuHV-1 = 1.65%, BHV-1 = 0.81%, EHV-1 = 0.79%, and EHV-4 = 0.16%), and lower values in the carnivore and VZV viruses (VZV = 0.136%, CHV-1 = 0.0056/0.020%, and FHV-1 = 0.0089%). It should be noted that for CHV-1, two distance values are given, and this is discussed below.

**Table 2:** Varicellovirus G + C content and intraspecies strain genetic distance

Virus	G + C %	Intrastrain distance %
BHV-1	72.6	0.77
BuHV-1	76.8%	NA
BHV-5	74.8	NA
EHV-1(Combined)	56.6	0.74
EHV-1 (Wild)	56.6	0.60
EHV-1 (Domestic)	56.6	0.14
EHV-3	68.1	NA
EHV-4	50.5	0.14
EHV-8	54.4	NA
EHV-9	56.1	NA
SuHV-1	73.6	1.23
FHV-1	45.8	0.004
CHV-1 (UK strains)	31.6	0.005
CHV-1 (Overall)	31.6	0.20
CeHV-9	40.5	NA
VZV	45.8	0.136

It is unclear if the higher genomic G + C content of the ungulate viruses is the result of genetic drift or evolutionary pressure. The observation that G + C content in varicelloviruses may be linked to intraspecies genetic distance may not be unprecedented, as G + C content appears to correlate with substitution rates in *Arabadopsis* [58]. It is highly unlikely that G + C content is the main driver of varicellovirus intraspecies genetic distance, however, it may be possible that G + C content is able to influence distance. Additional factors may influence intraspecies genetic variability in varicelloviruses, such as the propensity of the host to form large herds, transmissibility, and the number reactivation events in the life of the host. We also cannot eliminate the possibility that the genomic G + C content and intraspecies distance link is an artifact due to small sample size.

### Codon usage and mutational bias in the *Varicellovirus* genus

**Fig. 3:**
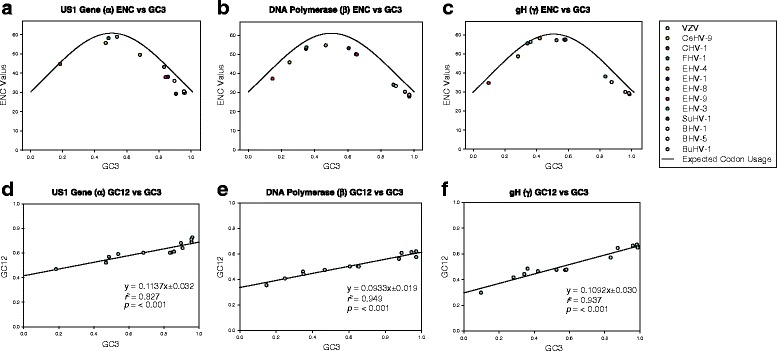
Effective codon number (ENC) – GC3s and Neutrality plots of the US1, UL30 polymerse, and UL22 (glycoprotein H) genes from the *Varicellovirus* genus. Figure 2a through c show the ENC-GC3s plots for the US1, UL30, and UL22 genes. For these plots, the ENC values for each of the three genes from each varicellovirus species were calculated and plotted against the G + C content of the synonymous third position. The black line represents the standard curve. The farther the plotted values are located from the standard curve, the influence of G + C mutation bias is implied to be lessened. A varicellovirus species key is shown to the right of the panels. Figure 2e, f, and g show the neutrality plots for the US1, UL30, and UL22 genes. The neutrality plots are constructed by plotting the average of the G + C content of the first and second codon positions (GC12), against the GC3s. Each plot point value represents a varicellovirus species, and includes a regression line. The *r,* ^2^ slope, and *p*-values are included in each graph

The observation of varying G + C content across the *Varicellovirus* genus lead us to investigate codon usage and mutational bias. Codon usage and mutational bias has been previously examined in other viruses such as canine parvovirus [35], Japanese encephalitis virus [37], and Zika virus [36]. For the present analysis, the effective codon number values of three genes, US1 (α), UL30 polymerase (β), and UL22 (glycoprotein H; γ) were calculated for each of the varicellovirus species (Table 3). These three genes were chosen to be representative of each kinetic class; α (immediate-early), β (early), and γ (late). ENC values range from 20 to 61, with 61 indicating no bias and 20 indicating extreme bias. The values show greater bias in all three genes from viruses that are either A-T or G-C rich (Table 3), for example CHV-1, SuHV-1, BHV-1, BHV-5, and BuHV-1. Next, ENC values in the context of mutational pressure were assessed by plotting the ENC values against the G + C content in the synonymous third codon position (GC3s), found in Fig. 3a, b, and c. The ENC plots for US1, UL30, and UL22 show that these three *Varicellovirus* genus genes are largely constrained by G + C mutation bias, as most data points are located close to the standard curve. Some of the data points are located farther away from the plot, such as SuHV-1 US1 (Fig. 3a), and EHV-4 (Fig. 3b), which suggest additional pressures influencing bias, possibly including natural selection.

**Table 3:** Effective codon number (ENC) values for the varicellovirus US1 (α), UL30 (β), and UL22 (γ) genes

Virus	US1	UL30	UL22
VZV	58.325	53.455	56.306
CeHV-9	55.196	46.375	48.746
CHV-1	44.246	37.827	34.744
FHV-1	57.692	54.214	55.561
EHV-4	48.973	55.309	58.247
EHV-1	37.591	50.632	57.499
EHV-8	42.716	53.786	57.220
EHV-9	37.448	50.471	57.474
EHV-3	29.358	34.598	38.156
SuHV-1	28.755	28.550	28.835
BHV-1	30.05	33.977	35.195
BHV-5	35.436	30.899	30.029
BuHV-1	29.317	29.396	29.430

Neutrality plots (GC12 vs GC3s) for US1, UL30, and UL22 were generated to further examine mutation and natural selection biases (Fig. 3c, d, and e). The neutrality plots showed significant results for US1 (*r* ^2^ = 0.827; *p* = < 0.001), UL30 (*r* ^2^ = 0.949; *p* = < 0.001), and UL22 (*r* ^2^ = 0.937; *p* = < 0.001), which confirmed mutational bias. The slopes for all three of the neutrality plots were shallow (US1 = 0.1137, UL30 = 0.0933, and UL22 = 0.1092), which indicated that mutation bias influenced codon usage only 11.37%, 9.33%, and 10.92% for each of these genes, respectively. ENC and neutrality plots appear to result in somewhat different conclusions in investigating codon usage and mutational pressure. Care should be taken in interpretation, as the analysis is genus wide, and not in a single species. G+C constrained mutation bias in varicelloviruses was confirmed as has been previously shown in vertebrate DNA viruses [39], however additional factors such as natural selection are likely to play major role as well.

### Bovine herpesvirus 1

**Fig. 4:**
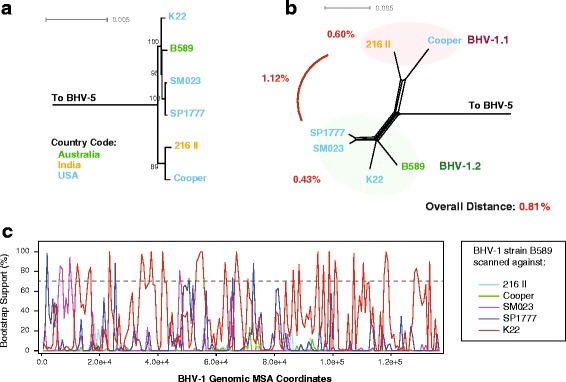
Phylogenetic, genetic distance, and recombination analysis of bovine herpesvirus 1 (BHV-1). **a** Maximum likelihood tree of BHV-1 genomic sequences generated using RAxML, with BHV-5 as an outgroup. Bootstrap values over 65% are shown. Phylogenetic network (**b**) was produced using Splitstree (kimura 2-parameter, gamma = 0.31376, and *p*-inverse = 0.45656). The genetic distance (Mega 6) between the two main BHV-1 clades (BHV-1.1 and BHV-1.2) was 1.12%. Viral strains are colored according to country of origin (green: Australia, orange: India, and light blue: USA). Recombination bootscan analysis (RDP4) of strain B589 scanned against the remaining BHV-1 strains is shown in panel **c**

BHV-1 has been traditionally divided into three subtypes; BHV-1.1, BHV-1.2a, and BHV-1.2b, with the 1.1 strains generally associated with IBR, and 1.2 with venereal disease phenotypes [59, 60]. It must be noted that strains of either type can cause respiratory and venereal disease phenotypes [61, 62], which suggests that genetic criteria may be a more reliable way to group BHV-1 strains than by clinical phenotype. To examine BHV-1 phylogeny, recombination, and genetic distance between clades, maximum likelihood trees and phylogenetic networks were generated, recombination bootscan analysis was conducted, and inter- as well as intra-clade distances were calculated. The ML tree and the phylogenetic network both recover two main clades (Fig. 4). Genetic distance analysis showed that the distance between the two groups was 1.12%, fulfilling the criteria for designating the clades 1.1 and 1.2. Thus, the BHV-1 clades retain the 1.x organization they had previously under our proposed nomenclature criteria. The genomic sequence analysis of BHV-1 recovered two main clades designated 1.1 and 1.2, however subclades within 1.2 were not detected. It is possible that as additional BHV-1 strains are sequenced, evidence of 1.2. subclades may become apparent. The overall genetic distance within BHV-1.1 was 0.60%, and within BHV-1.2 was 0.43% (Fig. 4b). Bootscan recombination analysis using BHV-1.2 strain B589 revealed extensive recombination from the remaining BHV-1.2 strains, but no significant recombinant signals from the BHV-1.1 viruses were detected (Fig. 4c). PHI recombination test analysis suggests that there is recombination in the dataset (*p* = <0.001; Table 1).

### Pseudorabies virus/SuHV-1

**Fig. 5:**
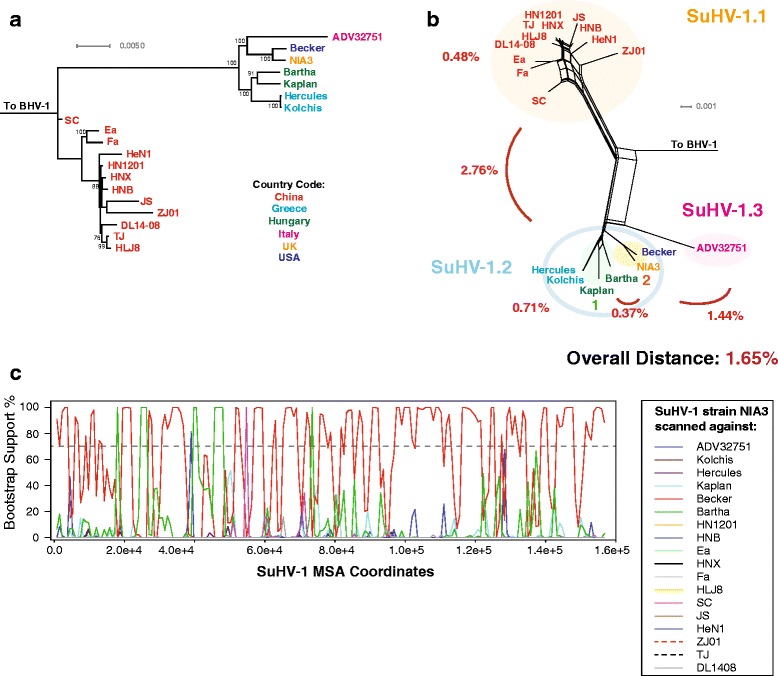
Phylogenetic, genetic distance, and recombination analysis of pseudorabies (SuHV-1). **a** Maximum likelihood tree of SuHV-1 genomic sequences generated using RAxML, with BHV-1 as an outgroup. Bootstrap values over 65% are shown. Phylogenetic network (**b**) was produced using Splitstree (kimura 2-parameter, gamma = 0.1520, and *p*-inverse = 0. 0.240). The genetic distance (Mega 6) between the Chinese (SuHV-1.1) and European/American (SuHV-1.2) clades was 2.76%. Recombination bootscan analysis (RDP4) of strain NAI3 scanned against the remaining SuHV-1 strains is shown in panel **c**. Viral strains in panels A and B are colored according to country of origin (**a**)

The phylogenetic structure of SuHV-1 strains was examined next. The genome based ML tree and phylogenetic network both recovered two basic clades, Chinese and European/American, which have been previously identified [63] (Fig. 5a and b). The phylogenetic network however, showed that the Italian domestic dog isolated strain ADV32751 was separated somewhat from the remaining European/American strains (Fig. 5b). Distance analysis showed that the genetic distance between the main Chinese and European/American clades was 2.76% (Fig. 5b). This comparatively large genetic distance between the two groups appears to be consistent with what is thought to be two independent domestication events, one in China [64], and another in modern day Turkey roughly 9000 years before present [65]. Additional calculations showed that the distance between strain ADV32751 and the remaining European/American strains was 1.44% (Fig. 5b). Based on the results of the distance calculations, we suggest that pseudorabies virus be designated as SuHV-1.1 (Chinese), SuHV-1.2 (main European/American), and provisional SuHV-1.3 (strain ADV32751) (Fig. 5b). It is unclear if the ADV32751 strain contains mutations which could have enhanced transmission to a domestic dog. Additionally, given the distance value with respect to the SuHV-1.2 viruses, the chance of genetic contributions from a European wild boar strain should not be excluded. Within the European/American SuHV-1.2 clade, two additional groupings were detected, and designated 1 and 2 based on the genetic distance (0.37%; Fig. 5b). A bootscan using SuHV-1.2 strain NIA3 against the remaining strains showed little to no recombination signals from either SuHV-1.1 or 1.3 (Fig. 5c). The lack of recombination signals between the SuHV-1.2 and 1.1 subclades is not unexpected due to geographic distances, however a recent report showed that the Chinese pseudorabies virus strain SC contained genomic contributions from the vaccine strain Bartha [30]. The PHI recombination test of all the SuHV-1 strains showed (Table 1) statistically significant recombination within the dataset (*p* = <0.001).

### EHV-1

**Fig. 6:**
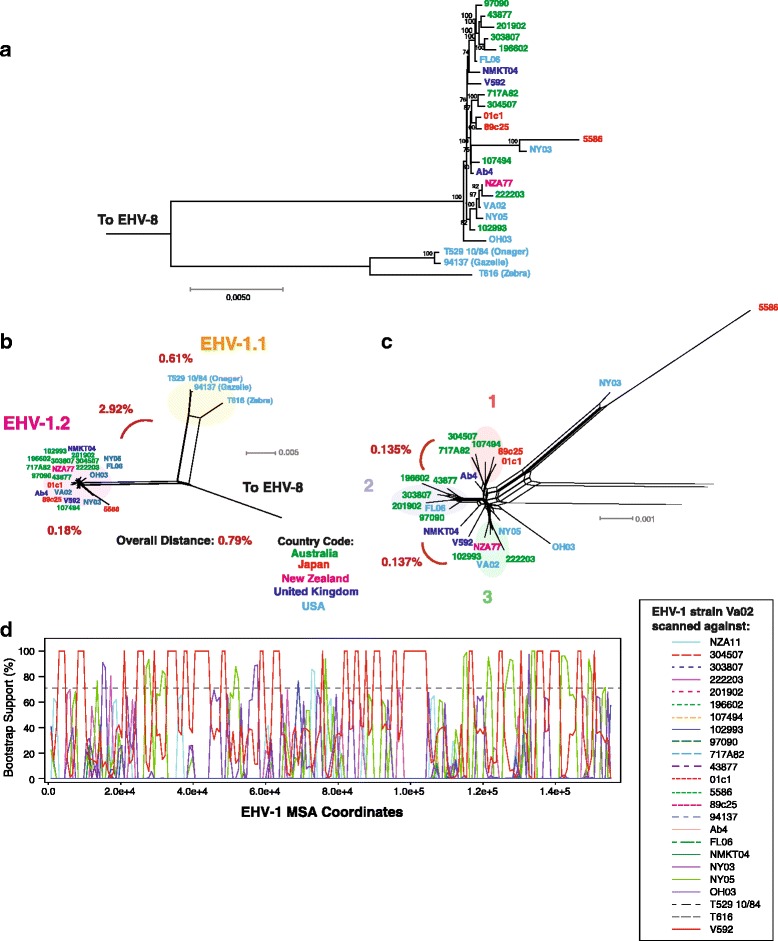
Phylogenetic, genetic distance, and recombination analysis of EHV-1. **a** Maximum likelihood tree of EHV-1 genomic sequences generated using RAxML, with EHV-8 as an outgroup. Bootstrap values over 65% are shown. Phylogenetic network (**b**) was produced using Splitstree (kimura 2-parameter, gamma = 1.0360, and *p*-inverse = 0.4940). The genetic distance (Mega 6) between the wild equine (EHV-1.1) and domestic horse (EHV-1.2) clades was 2.92%. (Panel **c**) A zoom of the domestic horse (EHV-1.2) strains from panel B shows one main grouping (**a**) and provisional B and C groups. Recombination bootscan analysis (RDP4) of strain Va02 scanned against the remaining EHV-1 strains is shown in Panel **d**. No recombination signals were detected from the EHV-1.1 strains. Viral strains in panels **a** and **b** are colored according to country of origin (Panel **c**)

The ML tree (Fig. 6a) shows a split between the wild and domestic horse derived EHV-1. The phylogenetic network also confirms this split (Fig. 6b). Genetic distance calculations resulted in 2.92% distance between the wild and domestic EHV-1 clades, and we suggest designating these EHV-1.1 (wild equine) and EHV-1.2 (domestic horse). The distance within the wild horse EHV-1.1 clade was higher than EHV-1.2, at 0.61% vs. 0.18% respectively. An expansion of the EHV-1.2 clade is shown in Fig. 5c, and shows three provisional clades, with clades 1 and 2 being 0.135% distant, and clades 2 and 3 0.137% distant. Two strains, NY03 and 5586, are separated from the remaining EVH-1.2 viruses and may represent a separate clade, however additional strains need to be islolated before this can be determined. EHV-1.2 strains NMKT04 and V592 occupy a position between clades 1 and 2 may be interclade recombinants. It is notable that EHV-1.2 strains do not appear to correlate to geographic origin, and may reflect the cosmopolitan nature of common breeds such as the Thoroughbred. Bootstrap recombination analysis scanning EHV-1.2 group 3 strain Va02 against the remaining strains showed no recombination from EHV-1.1 stains (Fig. 6c). When the EHV-1 strains were examined using the PHI recombination test (Table 1), statistically significant recombination was detected (*p* = <0.001). Wild equine derived EHV-1 strains cause severe infections, often neurological in both equine and non-equine captive animals [66–70], and some domestic horse EHV-1.2 strains can also cause myeloencephalopathy [71, 72]. A SNP (D752) within the polymerase gene of domestic horse viruses has been shown to influence the neurological disease phenotype, and is shared among wild equine strains [73, 74]. The genomic phylogenetic analysis (Fig. 5) of the domestic horse (EHV-1.2) strains did not sort the strains based on disease phenotype, i.e. neuropathology vs abortion (Fig. 6c). This finding along with data showing that non-D752 strains [75] can cause encephalitis strongly suggests multiple genes contribute to EHV-1 disease phenotypes, as has been observed in HSV-1 [76–79].

### EHV-4

**Fig. 7:**
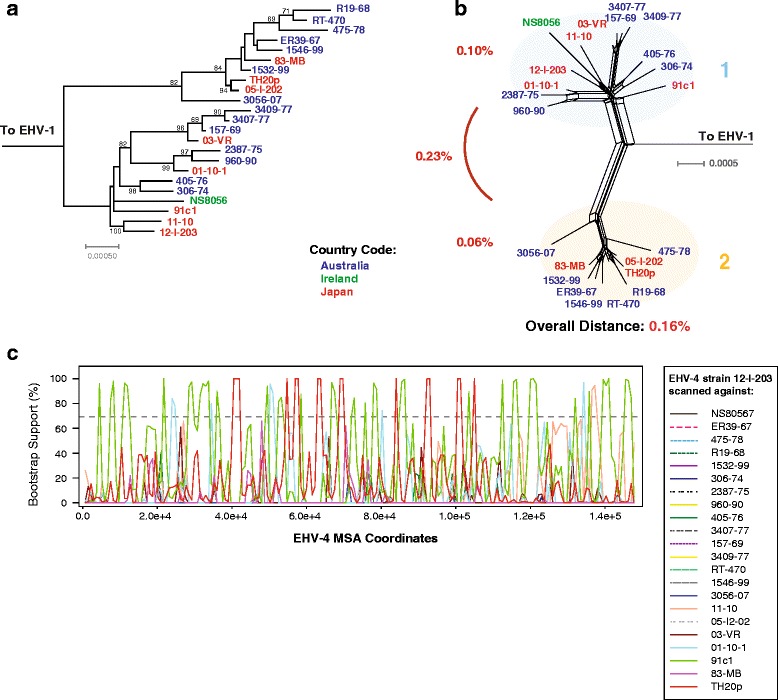
Phylogenetic, genetic distance, and recombination analysis of EHV-4. **a** Maximum likelihood tree of EHV-4 genomic sequences generated using RAxML, with EHV-1 as an outgroup. Bootstrap values over 65% are shown. Phylogenetic network (**b**) was produced using Splitstree (kimura 2-parameter, gamma = 0.8230, and *p*-inverse = 0.2770). The genetic distance (Mega 6) between group A and group B was 0.23%. Recombination bootscan analysis (RDP4) of strain 12-I-203 scanned against the remaining EHV-4 strains is shown in panel **c**. Viral strains in panels **a** and **b** are colored according to country of origin (Panel **a**)

Equine herpesvirus type 4 is an important equine disease, and causes rhinopneumonitis most commonly in foals [80], however it is not a reportable disease as is EHV-1. The phylogenetic ML tree (Fig. 7a) and phylogenetic network (Fig. 7b) of the available EHV-4 showed a split into two main groups (clade 1 and 2) as has been shown previously [32]. The genetic distance between the two clades was 0.23%. Both the ML tree and phylogenetic network showed that the EHV-4 viruses, like EHV-1, do not sort according to geographic origin and this is likely the result of the modern movement of common breeds globally. Recombination bootscan analysis (Fig. 7c) scanning EHV-4 group A strain 12-I-203 against the rest showed extensive recombination from both groups 1 and 2. Additional PHI recombination test analysis (Table 1) detected significant amounts of recombination in EHV-4 (*p* = <0.001).

### FHV-1

**Fig. 8:**
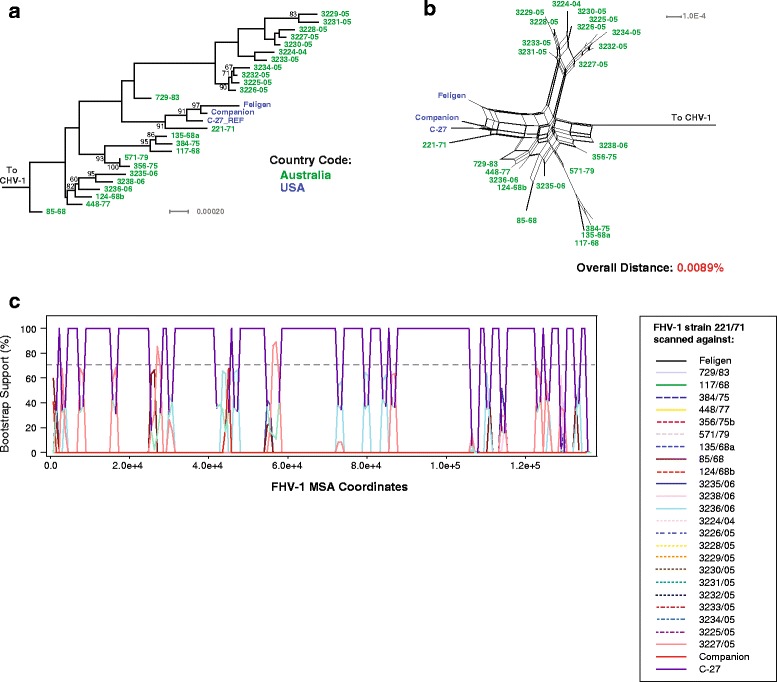
Phylogenetic, genetic distance, and recombination analysis of FHV-1. **a** Maximum likelihood tree of FHV-1 genomic sequences generated using RAxML, with CHV-1 as an outgroup. Bootstrap values over 65% are shown. Phylogenetic network (**b**) was produced using Splitstree (kimura 2-parameter, gamma = 1.37213, and no *p*-inverse value). The overall genetic distance (Mega 6, pairwise deletion) was 0.0089%. Recombination bootscan analysis (RDP4) of strain 221/71 scanned against the remaining FHV-1 strains is shown in panel **c**. Viral strains in panels **a** and **b** are colored according to country of origin (green: Australia, and blue: USA)

Feline herpes virus type 1 (FHV-1) is thought to be main cause of corneal ulceration [81] in cats, and can also contribute to upper respiratory disease [82]. Recently, several FHV-1 genomes from Australia were sequenced and genomic analysis did not reportedly detect recombination [83]. As part of our analysis of varicellovirus phylogenetic relationships, we analyzed the available FHV-1 genomic sequences. The ML tree and phylogenetic network (Fig. 8a and b) suggested some strain grouping, however, because the overall interstrain genetic distance is low (0.0089%), we did not designate any clades. Reticulations within the phylogenetic network implied the presence of recombination between the FHV-1 strains. Bootscan recombination analysis (Fig. 8c), as well the PHI recombination test (Table 1; *p* = <0.001) detected recombination signals. The difficulty in detecting recombination is likely due to the low interstrain genetic distance (0.0089%), and it is possible that as additional strains are sequenced, more recombination may be more readily identified. It would be surprising if recombination was not detected in FHV-1, as herpesviruses have been shown to be highly recombinagenic [29, 31, 84, 85].

### CHV-1

**Fig. 9:**
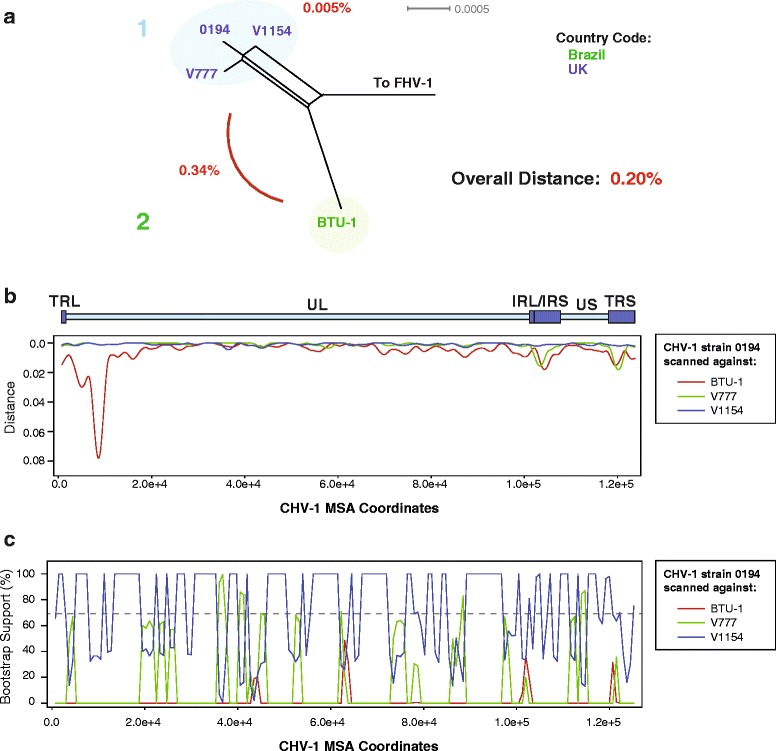
Phylogenetic, genetic distance, and recombination analysis of CHV-1. **a** Phylogenetic network was produced using Splitstree (kimura 2-parameter, gamma = 0.75049, and no *p*-inverse value). The genetic distance (Mega 6) between group A and group B was 0.34% (Panel **a**). A genomic similarity plot (RDP4, Panel B) was generated by scanning UK strain 0194 against the remaining CHV-1 strains. A schematic of the CHV-1 genome has been placed above the Simplot graph, with TRL = terminal repeat long, UL = unique long coding region, IRL = internal repeat long, IRS = internal repeat short, US = unique short coding region, and TRS = terminal repeat short. Recombination bootscan analysis (RDP4) of strain 0194 scanned against the remaining EHV-4 strains is shown in panel **c**. Viral strains in the phylogenetic network (**a**) are colored according to country of origin (green: Brazil, and blue: UK)

Until very recently, only three CHV-1 strains, collected between 1985 and 2000 from the UK had been sequenced [86], however a short time ago, a CHV-1 strain from Brazil was deposited into GenBank. The overall genetic distance between the three UK strains was very low, at 0.005% (Table 1; Fig. 9a). The CHV-1 strain from Brazil (strain BTU-1) was 0.34% distant from the three UK viruses, with an overall interstrain distance of 0.20% for the four viruses (Table 2; Fig. 9a). We performed a similarity plot using the MSA (multiple sequence alignment) without an outgroup, and found a deep trough at approximately 9 kb from the left end of the genome (Fig. 9b). The similarity trough corresponded to the UL50 deoxyuridine triphosphatase gene. Distance analysis comparing the UL50 protein sequences from the four CHV-1 viruses showed that the Brazilian BTU-1 strain UL50 was 12.2% distant compared to the UK strains (Table 4). Further blast searches (data not shown) determined that even though the BTU-1 strain UL50 protein sequence was 12.2% distant, it appeared closest to the remaining CHV-1 strains, rather than FHV-1. Bootscan recombination analysis using the UK derived 0194 strain as a reference only detected recombination signals from the other UK viruses, and none from BTU-1 (Fig. 9c). Curiously, the PHI recombination test did not detect statistically significant recombination (Table 1; *p* = 0.3082), and may be due to the small size of the dataset. Based on the data, we hypothesize that the BTU-1 strain may be the result of a recombination event between canine herpesvirus 1, and an unknown varicellovirus. It would be unlikely that positive selection would only affect a single gene in the virus to such a large extent (12.2% distance), however the possibility cannot be eliminated. Because the UL50 sequence most closely resembles the remaining CHV-1 viruses, it is likely that the unknown virus originated from an animal of the Caniformia suborder, which includes Brazilian species such as the maned wolf, bush dog, pampas fox, tayra, striped hog-nosed skunk, and crab-eating racoon.

**Table 4:** UL47 to UL54 maximum likelihood based protein distances of CHV-1 strain BTU-1 compared to UK derived viruses

Protein	Percent distance
UL47	0.78
UL48	0.23
UL49	0.12
UL49A	0
UL50	12.2
UL51	2.1
UL52	1.63
UL53	1.2
UL54	0

### Varicella zoster virus

**Fig. 10:**
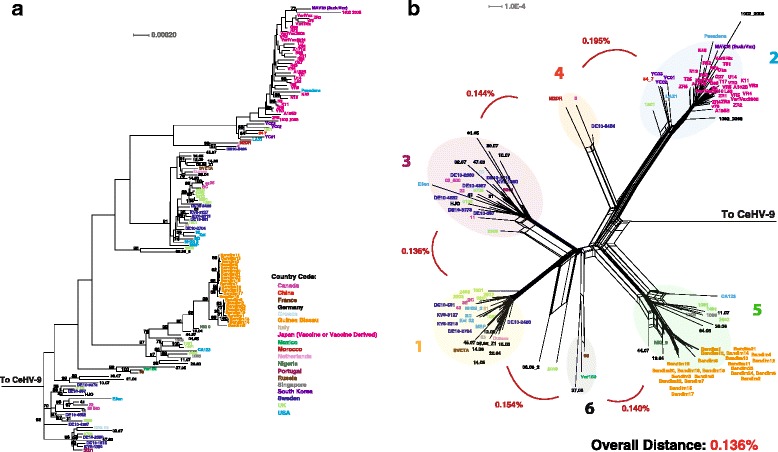
Phylogenetic, genetic distance, and recombination analysis of VZV. **a** Maximum likelihood tree of VZV genomic sequences generated using RAxML, with CeHV-9 as an outgroup. Bootstrap values over 65% are shown. A Phylogenetic network (**b**) was produced using Splitstree (kimura 2-parameter, gamma = 0.50800, and *p*-inverse = 0.19700). There are seven clades A-G, and the overall genetic distance (Mega 6, pairwise deletion) was 0.136%. Viral strains in panels **a** and **b** are colored according to country of origin (Panel **a**)

The Varicella-Zoster virus (VZV) causes chickenpox as well as shingles, and the phylogeny of VZV clades has been extensively studied [29]. We treated our analysis of VZV as an update, as additional strains have been sequenced and uploaded into GenBank. A ML tree and phylogenetic network using CeHV-9 (simian varicella virus) as the outgroup were constructed and are found in Fig. 10. The phylogenetic network suggests six clades, which are denoted numerically as described previously [29]. (Fig. 9b). PHI recombination analysis confirmed statistically significant recombination among the VZV strains (Table 1; *p* = <0.001%).

## Conclusions

In conclusion, this is the first genome based phylogenetic study of the entire *Varicellovirus* genus. In this study, we present a number of unique findings including results suggesting that a phylogenetic stricture exists between the ungulate viruses and the primate and carnivore viruses, a possible link between genome G + C content and intraspecies strain genetic diversity, the detection of recombination in all of the varicellovirus species including FHV-1, and that the Brazilian CHV-1 strain BTU may contain a genetic signal from an unknown varicellovirus in the UL50 gene. We also propose a clade nomenclature standardization for varicelloviruses. This work helps to deepen the understanding of varicellovirus genomics and evolution.

## Additional file

Additional file 1:
**Table S1.** Accession numbers for the sequences used in the present study. The species, isolation host, strain designation, genome size, country of isolation, isolation source, collection date, and accession number for each sequence is provided. **Table S2.** Unscaled *F* values. The the only reported *F* values were those which included cut-offs that result in two groups for all viruses. **Table S3.** Rescaled *F* values. The rescaled values of *F* simply indicates the relative size of *F.* The total value is on a scale from 0 to 3, with 3 meaning that the separation is as good as possible for all three viruses. (ZIP 42 kb)

## Abbreviations

AnHV-1: *Anatid herpes virus type 1*
BHV-1: *Bovine herpes virus type 1*
BHV-5: *Bovine herpes virus type 5*
BuHV-1: *Bubaline herpes virus type 1*
CeHV-9: *Cercopithecine herpes virus type 9*
CHV-1: *Canine herpes virus type 1*
EHV-1: *Equine herpes virus type 1*
EHV-3: *Equine herpes virus type 3*
EHV-4: *Equine herpes virus type 4*
EHV-8: *Equine herpes virus type 8*
EHV-9: *Equine herpes virus type 9*
ENC: Effective number of codons
FHV-1: *Feline herpesvirus type 1*
ICTV: International Committee on Taxonomy of Viruses
ML: Maximum likelihood
MSA: Multiple sequence alignment
NGS: Next-generation sequencing
PCV2: *Porcine circovirus type 2*
PHI: Pairwise homoplasty index
SuHV-1: Pseudorabies virus
USDA: United States Department of Agriculture
VZV: Varicella zoster virus
